# Recurrent Diffuse Pigmented Villonodular Synovitis in the Hand of a Pediatric Patient: A Case Report

**DOI:** 10.1016/j.jhsg.2023.01.013

**Published:** 2023-02-18

**Authors:** Gabriel Echegaray-Casalduc, David Deliz-Jimenez, Arnaldo SantaCruz-Casas, Christian Foy-Parrilla

**Affiliations:** ∗ Department of Orthopaedic Surgery, University of Puerto Rico, Medical Sciences Campus, San Juan, Puerto Rico; † Department of General Surgery, University of Puerto Rico, Medical Sciences Campus, San Juan, Puerto Rico

**Keywords:** Diffuse pigmented villonodular synovitis, Mass excision, Pediatric, Recurrence

## Abstract

Diffuse pigmented villonodular synovitis is characterized by synovial inflammation and hemosiderin deposition. It mainly occurs in adults, with the hip and knees being the most common sites of involvement. It is associated with high recurrence rates, with open synovectomy being the most common treatment method to avoid recurrence**s**. Few cases of diffuse pigmented villonodular synovitis have been reported in pediatric patients, especially in uncommon locations such as the hand. This case presents pathology-confirmed diffuse pigmented villonodular synovitis in the hand of a pediatric patient with multiple recurrences despite adequate surgical margins. The patient underwent mass excision with adjuvant radiation treatment after his last recurrence, with excellent functional outcomes and no recurrence at the five-year follow-up.

As reported in 1941 by Jaffe et al^1^, pigmented villonodular synovitis (PVNS) is a condition of the synovial membrane characterized by synovial inflammation and hemosiderin deposition in the synovium. On a microscopic level, the condition is characterized by lipid-laden macrophages, multinucleated giant cells, hemosiderin deposition, and both stromal and fibroblast cell proliferation.^2^

Pigmented villonodular synovitis was first introduced in 1852 as a neoplastic process because of its unrelenting growth pattern, capacity to erode surrounding bone and joint tissue, and high recurrence rate after resection.^3^ However, the pathology was later classified as chronic inflammation of the synovium, changing the focus from one of neoplastic origin to one based on inflammatory principles. This benign and recurrent condition tends to appear in patients between their third and fourth decade of life and is rarely seen in children; nevertheless, pediatric cases have been reported. Cytogenetic studies written by several authors suggest chromosomal involvement (trisomy seven and clonal DNA rearrangements) as a plausible explanation for the origin of this condition.^4^^,^^5^ Nevertheless, studies are yet to agree on the etiology of this disease process.

The most common sites of involvement are the knee; hip; and, to a lesser degree, the wrist, hand, and spine.^3^ Previously, PVNS was classified into two subtypes: diffuse PVNS (DPVNS) and localized PVNS. However, the latter is now considered a giant cell tumor of the tendon sheath (GCTTS), not PVNS. Apart from these technicalities, these two entities exist along a continuum of one disease process and, thus, have similar macroscopic and microscopic characteristics within the articulations and synovium they impact. Nonetheless, they differ behaviorally because DPVNS is more aggressive, presents a higher recurrence rate after wide surgical excision, and carries a poorer prognosis. Studies report a 25% recurrence rate for intra-articular disease and a 25% to 50% recurrence rate for extra-articular disease.^6^ Still, with careful and thorough surgical excision, recurrence rates as low as 8% have been reported.^2^ Diffuse PVNS is marked by slow and insidious pain, swelling, and eventually decreased ROM of the affected joint. With time, these repetitive insults to the affected joint(s) lead to severe articular cartilage destruction and eventual osteoarthritis.

There are a variety of surgical options to treat this condition. Open versus total arthroscopic synovectomy tends to be the most popular surgical approach. However, studies report that total open synovectomy is a more reasonable option when dealing with DPVNS.^7^ We present a case report of a pediatric patient with histologically confirmed DPVNS and multiple recurrences.

## Case Report

This is the case of a 14-year-old boy who presented to our clinic with a chief complaint of a left hand mass. Physical examination of the left hand noted a distal tip mass of the left fifth finger. Radiographs of the fifth digit showed a soft tissue bulge lateral to the distal phalanx’s radial aspect, correlating with clinical findings.

High-resolution sonography of the left hand demonstrated a soft tissue heterogeneously hypoechoic mass at the tip of the fifth digit with peripheral increased vascularity. The mass involved the DIP joint synovium, flexor, and extensor tendons. The patient underwent mass excision and flexor tenolysis. The mass in the left fifth finger was located at the distal, volar, and radial aspect and was adherent to the flexor digitorum profundus and radial digital nerve. The mass was hard, multilobulated, tan-yellow, and rubbery, suggesting DPVNS. After surgery, the patient regained full ROM with no loss of sensation.

**Figure 1:**
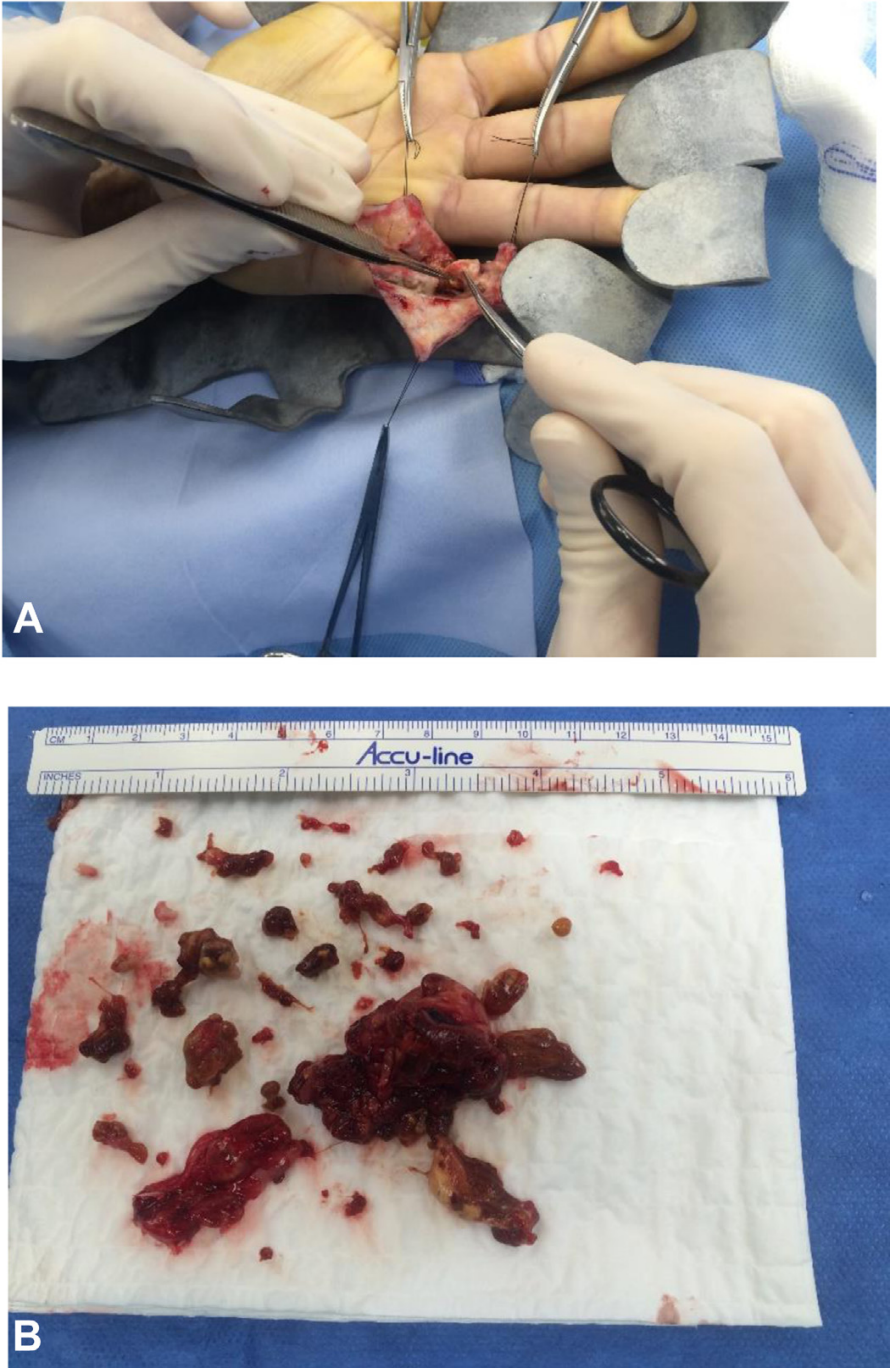
Excision of first mass recurrence. **A** Intraoperative excision of mass. **B** Excised mass.

**Figure 2:**
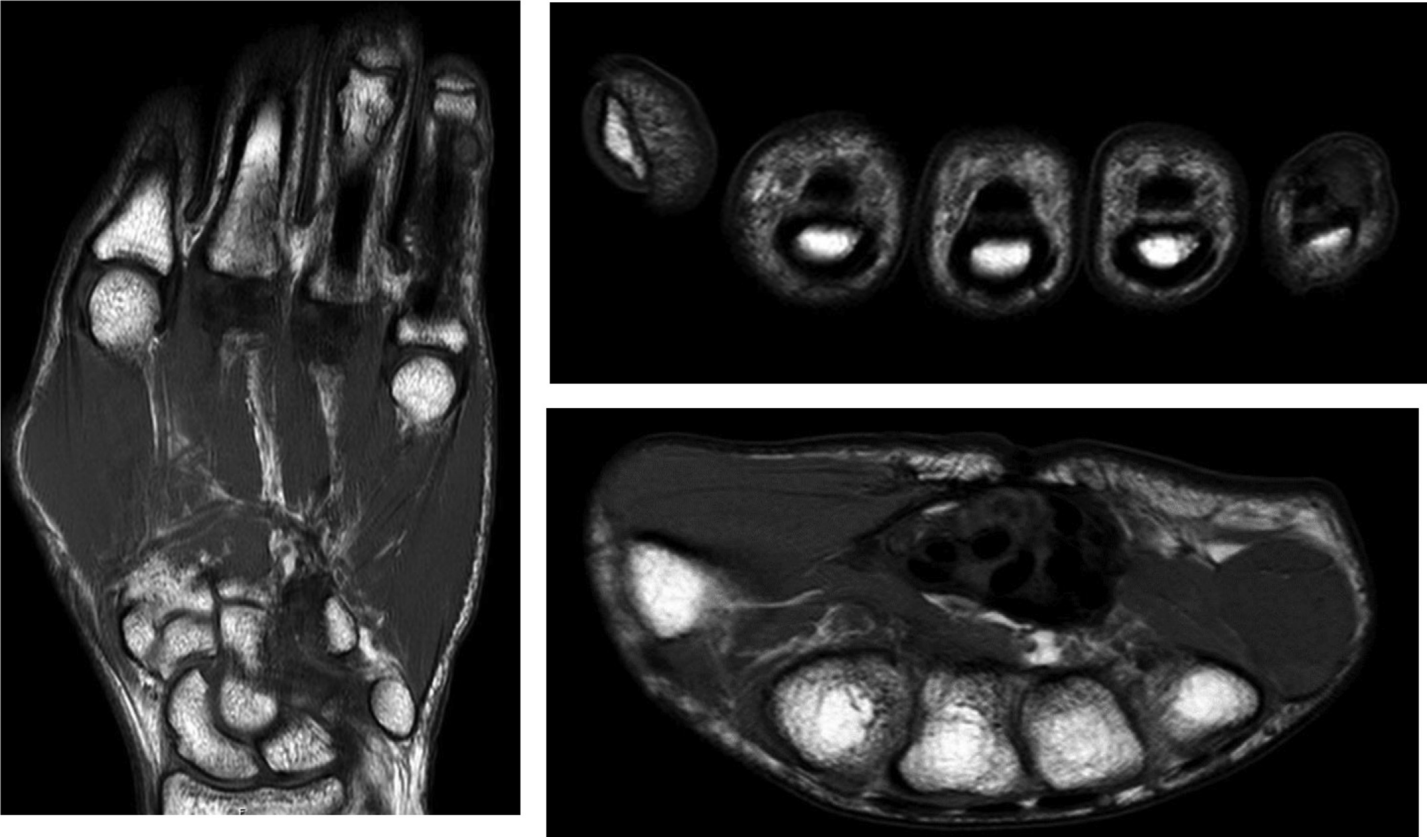
Magnetic resonance imaging findings of second mass recurrence.

However, 2 years after this initial mass excision, the patient returned to the clinic with a recurrence of the mass. The patient returned to the operating room for excision of the mass recurrence and again, the diagnosis of DPVNS was pathologically confirmed (Fig. 1). The mass was removed piecemeal because it was an extensive lesion within the neurovascular bundle, which prohibited en bloc resection. On follow-up examination, the patient had regained full ROM with intact sensation and no pain. The patient continued his uneventful recovery until 1 year later, when he returned to the clinic with yet another recurrence of the soft tissue mass (Fig. 2).

**Figure 3:**
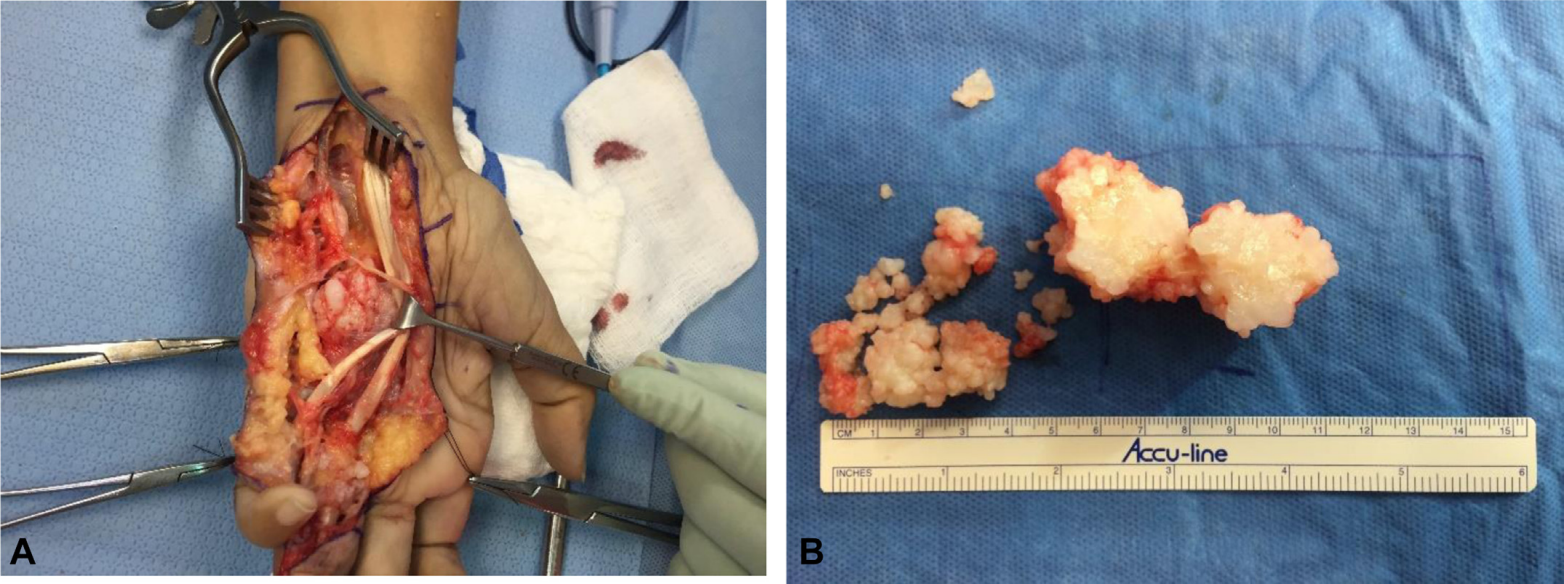
Excision of second mass recurrence. **A** Intraoperative view of mass. **B** Excised mass.

The patient presented with a more proximal, aggressive mass in the ulnar, palmar side of the hand. On this occasion, the oncology service was consulted, adjuvant radiation was given, and surgery was performed to remove soft tissue mass (Fig. 3). In this second recurrence, the surgical exposure was more extensive because of the highly aggressive nature of the mass and its more proximal localization. Additionally, a more extensive surgical exposure was preferred to allow an approach to the mass through native tissue rather than scar tissue. The pathology again confirmed the diagnosis of DPVNS. There was no recurrence of the mass at the five-year follow-up, and clinical findings reveal a full ROM and no pain or loss of sensation in the hand.

## Discussion

Giant cell tumors of the synovial and tendon sheaths can be classified into two forms: localized (giant cell tumors of the tendon sheath or nodular tenosynovitis) and diffuse (diffuse-type giant cell tumors or PVNS). Tumors of localized form are confined to a distinct area of the synovium, whereas tumors of the diffuse form demonstrate extensive involvement of the whole synovial membrane and capsule.^4^^,^^6^ Diffuse PVNS and GCTTS can be differentiated from each other by histological findings of hemosiderin deposits throughout the synovium, foam cells, and multinucleated giant cells.^6^ Diffuse PVNS are described in histopathology studies as infiltrative lesions with fewer osteoclastic giant cells and small histiocyte-like cells covered by one or more layers of synovial cells. Rounded cells with hemosiderin granules are also observed, and the lesions are more hypercellular with fewer multinucleated giant cells compared to GCTTS.^4^

Clinical differences between DPVNS and GCTTS lesions are also noted. Their symptoms vary depending on the location and size of the lesion, associated diseases, and history of trauma.^6^ Joint destruction, arthritis, and symptom sequels are more prominent in DPVNS.^3^ The most critical clinical difference is the incidence of recurrence after excision. Diffuse PVNS has a recurrence probability of 18% to 46%, whereas GCTTS has a recurrence probability of 8% to 29%.^4^ In this patient, a highly aggressive, recurrent lesion was observed, requiring multiple surgical excisions and the addition of adjuvant radiotherapy. Studies have reported excellent outcomes and decreased recurrence rates with adjuvant radiotherapy after open excision.^8^^,^^9^ Therefore, given the multiple recurrences in this patient and studies supporting adjuvant radiotherapy to avoid recurrence**s**, radiotherapy was included in the patient’s management. Potential adverse effects from radiotherapy that must be considered include toxicity, skin changes, joint stiffness, and radiation-induced sarcoma. However, the patient history suggested that the benefits outweighed the potential complications. Recurrence is due to incomplete excision of the tumor or the presence of an unidentified satellite lesion and may be predictable on the basis of specific patterns of tissue involvement. Given the excellent outcomes following excision with the addition of radiotherapy, we recommend radiation therapy early, especially in extensive, aggressive masses. This may prevent repeat surgical procedures in a pediatric patient’s hand that may contribute to excess scarring and flexor contractures. Factors related to the degree of recurrence include involvement of the joint capsule, extensor tendon, and flexor tendon.^10^

There are multiple described cases of both GCTTS and PVNS in adults. However, only a few cases have been reported in the pediatric population and they are limited to the knee and lower extremities. The hand and wrist were the least likely locations for its appearance in pediatric patients, and none had the multiple recurrences observed in this case. Kay et al^11^ described one case of a pediatric patient with diffuse pigmented villonodular tendon synovitis of the wrist. In this case, the dorsal wrist mass was excised with no same-site recurrence.^11^ However, a year and a half after the wrist lesion was removed, the otherwise healthy patient developed a DPVNS lesion in the knee, which was removed with no subsequent recurrence. Our case was different, given the multiple same-site recurrences in an atypical location. However, after adding adjuvant radiotherapy, which had been used for DPVNS recurrences in adults, our patient did not have a mass recurrence at the five-year follow-up.

## Informed Consent

Written informed consent was obtained from the patient for the publication of this case report and accompanying images according to the CARE guidelines.
